# Inactive yet indispensable: the tale of Jarid2

**DOI:** 10.1016/j.tcb.2010.10.004

**Published:** 2011-02

**Authors:** David Landeira, Amanda G. Fisher

**Affiliations:** Lymphocyte Development Group, MRC Clinical Sciences Centre, Imperial College School of Medicine, Hammersmith Hospital Campus, Du Cane Road, London, W12 0NN, UK

## Abstract

Methylation of histone tails is believed to be important for the establishment and inheritance of gene expression programs during development. Jarid2/Jumonji is the founding member of a family of chromatin modifiers with histone demethylase activity. Although Jarid2 contains amino acid substitutions that are thought to abolish its catalytic activity, it is essential for the development of multiple organs in mice. Recent studies have shown that Jarid2 is a component of the polycomb repressive complex 2 and is required for embryonic stem (ES) cell differentiation. Here, we discuss current literature on the function of Jarid2 and hypothesize that defects resulting from Jarid2 deficiency arise from a failure to correctly prime genes in ES cells that are required for later stages in development.

## Introduction

In eukaryotic cells, DNA is wrapped around nucleosomes composed of an octamer of the core histones H2A, H2B, H3 and H4. Linker histone H1 can facilitate further packaging of nucleosomes into a higher-order chromatin structure. Histone tails that extrude from the core octamer can be post-translationally modified by a number of different processes, including methylation, acetylation, phosphorylation, ubiquitylation and SUMOylation. Methylation targets lysine and arginine residues and these modifications can have a profound impact on the regulation and inheritance of transcriptional programs. Histone methylation is thought to be important for activating gene expression (*e.g.*, trimethylation of lysine 4 on histone H3 [H3K4me3]) and for silencing gene activity (*e.g.*, trimethylation of lysine 27 on histone H3 [H3K27me3]). Historically, methylation of histones was considered to be a relatively stable modification, in contrast to histone acetylation. However, in the last few years many enzymes capable of antagonizing the addition of methyl groups or of directly removing methyl groups from histones have been identified [1]. The largest family of histone demethylases identified so far is the Jumonji family, of which the founding member is Jarid2/Jumonji [2]. These proteins contain a Jumonji C (JmjC) domain that is believed to catalyze histone demethylation. In the case of Jarid2, alterations in the proposed catalytic site at the core of the JmjC domain are predicted to impair its catalytic activity [2], and yet Jarid2 is apparently indispensable for normal development.

Jarid2 has pleiotropic and essential roles in mouse development [3,4]. The molecular mechanism by which this inactive histone demethylase modulates mammalian development is not well understood, but recent studies have shown that Jarid2 associates with polycomb group (PcG) proteins in several cell types, including embryonic stem (ES) cells, thymocytes, HeLa and HEK 293T fibroblasts, and is critical for ES cell differentiation [5–9]. PcG proteins were identified more than 30 years ago as regulators of *Hox* genes and development in *Drosophila melanogaster* [10,11] and are currently thought to regulate gene expression during development and patterning in many organisms [12,13]. PcG proteins also have a critical role in adult tissue homeostasis because abnormal expression can promote tumor formation and cancer in various tissues [13,14]. Here, we examine current knowledge about the role of Jarid2 in mammals and discuss how the recently reported interaction of Jarid2 with PcG proteins is critical for preparing ES cells for differentiation by facilitating the transcriptional priming of developmental regulator genes. This proposed role for Jarid2 might provide an explanation of the reported pleiotropic effects of Jarid2 removal during embryogenesis.

## The Jumonji family of histone demethylases

**Figure 1:**
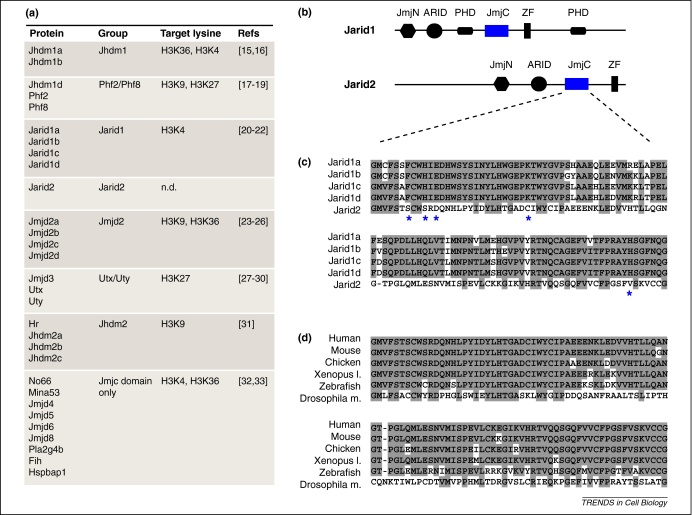
Jarid2 belongs to the JmjC-domain-containing family of proteins. **(a)** Several proteins that contain a JmjC domain catalyze the removal of methyl groups from lysine residues at specific positions on histone H3, as indicated. n.d., not determined. **(b)** Comparison of functional domains within the Jarid2 and Jarid1 proteins: Jumonji N (JmjN), AT-rich interaction domain (ARID), plant homeodomain zinc finger domain (PHD), Jumonji C (JmjC) and C5HC2 zinc finger (ZF). In addition, the N terminus of Jarid2 contains a nuclear localization sequence (aa 1–131) and a *trans*-repression domain (aa 132–222). **(c)** Amino acid sequence alignment of the JmjC domain of mouse Jarid1 and Jarid2 proteins. Asterisks indicate the amino acids that are critical for cofactor (iron and α-ketoglutarate) binding. Conserved amino acids are highlighted in grey. **(d)** Alignment of the amino acid sequence of the JmjC domains of Jarid2 proteins in different organisms; conserved residues are highlighted in grey.

Jarid2 contains a JmjC domain and has extensive homology to a large group of evolutionarily conserved proteins known as the Jumonji family [2]. Proteins in this family each contain a JmjC domain that is important for catalyzing the removal of methyl groups from specific lysine residues on histones through an oxidative reaction that requires iron and α-ketoglutarate as cofactors. Jumonji proteins demethylate lysines in histone H3 at positions 4, 9, 27 and 36 (Figure 1a) [15–33]. Jarid2 shares the highest degree of homology with Jarid1 proteins, which are capable of recognizing and removing methyl groups from di- (me2) and trimethylated (me3) histone H3K4 (Figure 1a) [20–22]. Jarid1 contains at least six functional domains (Figure 1b), including two plant homeodomain (PHD) zinc-finger domains that are not present in Jarid2 [2]. Both proteins contain Jumonji N (JmjN), AT-rich interaction domain (ARID), and a zinc finger (ZF), in addition to JmjC. In the case of Jarid2, however, alterations in the underlying amino acid sequence within the predicted cofactor binding site at the core of the JmjC domain are predicted to abolish or severely impair the histone demethylase activity of the resulting protein (Figure 1c, asterisks) [2] and no demethylating activity for this protein has been demonstrated to date. In view of this, it is perhaps surprising that the altered JmjC domain contained within Jarid2 is highly conserved (Figure 1d), a result that suggests that Jarid2 has a function that might be independent of histone demethylation. Although there are currently no biochemical data to support an alternative role for Jarid2, it has been claimed that its ARID and ZF domains directly bind DNA [8,34,35], albeit with low affinity [36]. In addition to these conserved domains, the N terminus of Jarid2 contains a nuclear localization sequence (Figure 1b) and a *trans*-repression domain that is required for repressor activity in Gal4 reporter systems [7,34].

## Jarid2 in the developing embryo

*Jarid2/Jumonji* was identified in 1995 as a regulator of neural development, in a gene trap mutagenesis screen in mice [37]. It was named *Jumonji* because of the cruciform shape of neural grooves (Jumonji means cruciform in Japanese) observed in *Jarid2*-targeted embryos. Subsequent studies showed that *Jarid2* is expressed by a restricted population of cells within the developing mouse brain, heart, spinal cord, liver and thymus [3,4]. Jarid2-deficiency resulted in a range of phenotypes, the severity and developmental onset of which were dependent on the genetic background against which chimeras were made and backcrossed (Table 1). For example, embryos generated from targeted ES cells [37] showed defects in neural tube formation [37], neural and heart development [38–42] or hypoplasia of liver, thymus and spleen, together with impaired definitive hematopoiesis [38,43,44]. Embryo lethality ensues between 10.5 and 15.5 days of gestation, according to strain, and is multi-factorial. The availability of new, independently derived gene-trap lines [45,46] and ES cells containing *Jarid2* floxed alleles [6,9,47] will facilitate verification and systematic examination of these complex phenotypes and investigation of the mechanisms that partially compensate for Jarid2 loss *in vivo*. Genetic analyses have also shown that mutations in the *JARID2* gene are associated with several congenital defects in humans, including nonsyndromic cleft lip [48], spina bifida and congenital heart defects [49], as well as with schizophrenia [50,51]. JARID2 is expressed by cells in the adult brain and heart in humans [52,53], mimicking previous observations in mouse adult heart, skeletal muscle, kidney, brain, and thymus [3,4]. Although we do not currently understand the basis of the association of *JARID2* mutation with these human diseases, collectively these data underscore the pleiotropic role of JARID2 in the development and homeostasis of many tissues.

## Jarid2 in the developing heart: a critical regulator of cell proliferation?

The role of Jarid2 in the developing embryo is not well understood. Most studies to date have examined abnormalities arising in the developing heart and nervous system in *Jarid2*-targeted embryos. It has been suggested that Jarid2 modulates cardiomyocyte proliferation and coordinates cell cycle exit during neurogenesis by directly repressing transcription of cyclin D1 [3,40,42]. Experiments in which Flag-tagged Jarid2 was over-expressed in fibroblasts have suggested that Jarid2 promotes the recruitment of histone methyltransferases (G9a and GLP) to, and subsequent H3K9 methylation of, the cyclin D1 promoter *in vitro* [54] (Table 2). Although this provides a plausible model of the action of Jarid2 in regulating proliferation in the heart, it is not yet known whether endogenous Jarid2, G9a and GLP interact directly in primary cardiomyocytes, and double-mutant embryos lacking both Jarid2 and cyclin D1 do not show complete reversion to a wild-type phenotype [40]. It has also been proposed that Jarid2 modulates the activity of other transcription factors and cell-cycle regulators (Table 2), including Gata4 [55], Rb protein [56], MEF2A [57], Zfp496 [58] and Nkx2.5 [55], a cardiac-associated regulator that represses Jarid2 expression in the heart [59].

In mice, the absence of Jarid2 increases proliferation of cardiomyocytes [40], megakaryocytes [60], fibroblasts [61,62] and cells within the developing brain [42]. At the same time, differentiation of cardiomyocytes [39] and hepatocytes [63] is partially impaired in *Jarid2*-targeted embryos. This suggests that Jarid2 is important for maintaining the correct balance between proliferation and differentiation, and that in some circumstances *Jarid2* might act as a tumor suppressor gene. This conjecture is supported by findings showing that Jarid2 expression is downregulated by the oncogenic microRNA *miR-155* in B-cell tumors [61] and that *Jarid2* is a candidate in genetic screens for factors that override senescence (J. Gil, personal communication).

## Jarid2 is a PRC2 component in ES cells and is required for gene priming

ES cells are derived from the inner cell mass of pre-implantation blastocysts, from which all the cells of the adult soma are derived. These cells retain a potential to self-renew and to differentiate into the three germ layers under appropriate conditions and therefore are classed as pluripotent. Although Jarid2 has been regularly listed as a key component in transcriptional networks that underlie pluripotency in both mouse and human ES cells [64–68], the nature of the role of Jarid2 in pluripotency has, until recently, been unclear. Recently, several groups reported that Jarid2 associates with PcG proteins in ES cells and that Jarid2 is important for successful ES cell differentiation (K.P. Yan and R. Shiekhattar, personal communication) [5–9].

**Figure 2:**
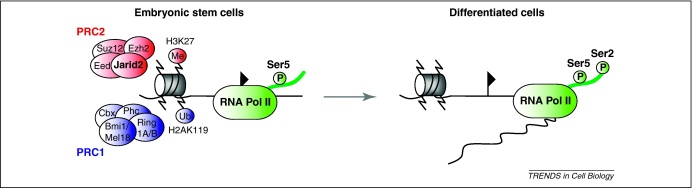
Jarid2 is required for Ser5-phosphorylated RNAPII recruitment to bivalent domains in undifferentiated mouse ES cells. In pluripotent ES cells, establishment of primed chromatin involves the recruitment of Ser5-phosphorylated RNAPII (green) to the promoter regions (flag) of PRC-repressed target genes. PRC2 subunits (red) methylate H3K27, providing a docking site for PRC1 and Ring1A/B-mediated mono-ubiquitination of H2A (blue). Loss of PRC-mediated repression on ES cell differentiation results in productive gene expression. In *Jarid2* null ES cells, Ser5-phosphorylated RNAPII is not efficiently recruited to PRC-repressed genes. This lack of priming results in the failure of mutant cells to efficiently express target genes when induced to differentiate.

PcG proteins regulate gene expression during embryonic development [12,13] and form multi-component complexes that are capable of modifying histone tails within chromatin. Polycomb repressor complexes (PRCs) act sequentially, and a widely accepted model suggests that PRC2 (which contains the core subunits Eed, Suz12 and Ezh2) methylates H3K27 (H3K27me3) through its catalytic subunit Ezh2. PRC1 (which contains the subunits Ring1A and/or Ring1B, Bmi1 and/or Mel18, Cbx subunits and Phc subunits) binds H3K27me3 and catalyzes the mono-ubiquitination of lysine 119 on H2A (H2AK119ub1) through the E3 protein ligase subunits Ring1A and Ring1B (Figure 2) [12,69]. It has been shown that PRC1 recruitment at certain loci occurs independently of H3K27me3 [12,70], which suggests that these recruitment events can be uncoupled. Recruitment of PRC can in turn lead to transcriptional repression of selected target genes by mechanisms that are not fully understood, but are likely to involve both physical compaction and the inhibition of efficient transcriptional elongation by locally bound RNA polymerase II (RNAPII). In ES cells, PRC1 and PRC2 binding is enriched at the promoters of hundreds of genes, many of which encode transcription factors that are activated as ES cells differentiate and have important roles in development [71–76]. The promoters of these genes have not only H3K27me3 in ES cells, but also histone marks that are associated with transcriptionally active domains, including H3K4me3 [73,77]. This opposing configuration, often referred to as bivalent chromatin, has been detected in ES and other cell types [78] and is characterized by the presence of bound RNAPII in which Ser5-phosphorylated (the initiating form of RNAPII), rather than Ser2-phosphorylated forms (characteristic of elongating RNAPII) predominate (Figure 2) [74,79]. On the basis of these observations, we and others have proposed that the selective priming of developmental regulator genes in ES cells (co-enriched for RNAPII and PRCs) might be functionally relevant in enabling these cells to execute different lineage programs in response to specific cues [80–82]. In other words, genes for which RNAPII is already loaded (but restrained from generating mature full-length transcripts because of bound PRC) might be primed for fast and efficient activation in response to differentiation-mediated PRC withdrawal.

In the last year, several studies have provided compelling evidence that Jarid2 is a component of PRC2 in ES cells and that Jarid2 and PRC2 bind to a largely overlapping set of target genes (>90% in common genome-wide). Jarid2 seems to be required for efficient binding of PRC2 and PRC1 to target genes [5–9], although ChIP analysis in Jarid2 knockdown [5,7,8] or knockout ES cells [6,9] did not show a consistent change in H3K27 methylation levels; in three studies, H3K27me3 decreased following Jarid2 depletion [7–9], whereas loss of Jarid2 resulted in either no change [5] or enhanced H3K27me3 at similar target genes [6] in other studies. This apparent lack of consensus suggests that although reduced binding of PRC2 to target genes is a feature of Jarid2 depletion, this might have variable consequences for H3K27me3 levels. The interpretation by Orkin and Wysocka is that Jarid2 acts as a molecular rheostat and fine-tunes PRC2 functions by interfering with H3K27 methyltransferase activity [5,6,83]. Conversely, Reinberg and Helin claim that Jarid2 is important primarily for targeting PRC2 to target genes, but can also stimulate H3K27 methyltransferase activity [7,8]. *In vitro* experiments using nucleosomes and recombinant PRC2 and Jarid2 proteins have failed to show any consistency; Jarid2 can stimulate [8] or inhibit [5,6] the methyltransferase activity of PRC2 towards H3K27. One explanation that might reconcile these opposing views is that binding of Ezh2 to PRC2 target genes is diminished but not abolished in *Jarid2*^−/−^ cells, such that the activity of remaining Ezh2 is sufficient to maintain H3K27me3 levels, at least at certain genes. Moreover, compensatory mechanisms, perhaps involving the redundant H3K27 methyltransferase Ezh1 [76], could also be operating in Jarid2-depleted cells. The fact that some Ezh2, Suz12 and Eed is still selectively recruited to PRC2 targets in *Jarid2*^−/−^ ES cells [6,9] confirms that PRC2 can be recruited in a Jarid2-independent manner.

Although discrepancies in H3K27me3 levels in the various Jarid2-deficient cell lines analyzed remain unresolved, the fact that the effects of Jarid2 removal on H3K27me3 levels are modest makes it unlikely that they could fully account for the phenotypic abnormalities of *Jarid2*^−/−^ ES cells – namely, grossly impaired ability to differentiate and increased proliferation. In addition, *Jarid2*^−/−^ ES cells do not display the genome-wide derepression of PRC2 target genes that is characteristic of the withdrawal of other PRC2 components, including Eed, Ezh2 and Suz12 [71,73,75,76]. If anything, PRC2 target gene expression was lower in *Jarid2*^−/−^ ES cells than in controls [6,9]. This suggests that Jarid2 does not function as a conventional core repressive component in PRC2, unlike Eed, Suz12 and Ezh2. In agreement with this, *Jarid2*^−/−^ ES cells reprogrammed somatic cells efficiently in heterokaryon reprogramming assays, whereas ES cells lacking Eed, Suz12 or Ezh2 fail to reprogram somatic cells because they inappropriately express PRC2-regulated factors that interfere with conversion to pluripotency [84].

A clue to the role of Jarid2 came with the discovery that bivalent domains in *Jarid2*^−/−^ ES cells lacked Ser5-phosphorylated RNAPII enrichment [9]. It has been proposed that this form of RNAPII characterizes primed genes before productive transcription. When induced to differentiate to mesoderm, endoderm or neural ectoderm, *Jarid2*^−/−^ ES cells were unable to initiate gene expression programs associated with each differentiation pathway and did not elicit the productive transcription of PRC2 target genes [6–9]. Collectively, these results suggest that Jarid2 is required to establish or maintain Ser5-phosphorylated RNAPII at bivalent/PRC2-repressed domains in ES cells and, more importantly, that priming of this subset of genes is important for ES cell function (Figure 2).

## Conclusions and future perspectives

Jarid2 has previously been implicated as a regulator of embryonic development through a mechanism thought to involve Jarid2-mediated recruitment of H3K9 methyltransferase complexes (G9a and GLP) to the cyclin D1 promoter [54]. Recent studies now indicate that Jarid2 associates with the H3K27 histone methyltransferase complex PRC2 in undifferentiated ES cells, which regulates myriad developmental regulator genes required for subsequent ES cell differentiation. Jarid2-deficient ES cells differentiate inefficiently *in vitro*, which suggests that Jarid2 is required for execution of the very early stages of development *in vivo*. However, *Jarid2*-targeted embryos develop through gastrulation and show pleiotropic defects only later during organogenesis. This apparent contradiction of when and where Jarid2 acts *in vitro* versus *in vivo* might have a number of different explanations. First, it is possible that leaky expression from gene-trapped *Jarid2* alleles might occur in targeted embryos, or that maternally derived *Jarid2* transcripts provide a significant contribution to the developing embryo. Second, any requirement for Jarid2 might be selectively compensated by other factors or signaling pathways *in vivo*, at least up to late gastrulation stages. Alternatively, it is possible that Jarid2 is only required to enable *in vitro* cultured ES cells to successfully escape their proliferative cycle and exit from self-renewal. Although we cannot rule out these possibilities at present, it seems unlikely that the role of Jarid2 in early development is limited to ES cells, because it is also required for successful gastrulation in *Xenopus laevis* [5]. Maternal contribution of *Jarid2* transcripts is worth considering because JARID2 is expressed in human mature oocytes [85] and its role in *Xenopus* gastrulation has been assayed using Jarid2 morpholinos that would block maternal transcripts [5]. In addition, the variation in phenotypes observed when *Jarid2*-targeted mice are bred onto different genetic backgrounds supports the idea that compensatory mechanisms *in vivo* could effectively mask a role for Jarid2 in early development*.* Finally, it is perhaps worth pointing out that self-renewing pluripotent cells *in vivo* are transient and limited to a very tight window in development (blastocyst stage) [86]. In the case of ES cells, for which pluripotent self-renewal has been artificially prolonged, it might be far harder for compensatory mechanisms, such as Ezh1, to operate successfully. This might explain why embryos lacking Jarid2 successfully develop right up to gastrulation whereas *Jarid2*^−/−^ ES cells show more pronounced differentiation defects.

The capacity of ES cells to self-renew and give rise to progeny for many different cell types is not yet understood at the molecular level. The discovery of so-called bivalent chromatin domains, for which the transcriptional machinery and PRC1 and PRC2 complexes are co-recruited to a cohort of non-expressed but primed genes, might provide some important insights into how genomic flexibility is underwritten. This process seems to require Jarid2 and enables cells to efficiently transit from pluripotent to differentiated states. Although there is still much to do, particularly in understanding the molecular relationship between Jarid2, PRC and Ser5-phosphorylated RNAPII, it is likely that this work will further our understanding of how ES cells retain their capacity to self-renew and differentiate into multiple lineages.

## Figures and Tables

**Table 1:** Morphological abnormalities in Jarid2-deficient mice

Mutagenesis strategy	Mouse strain	Lethality	Developmental abnormality	Refs
Gene trap (intron 2) in E14 ES cells (transfection)	Mixed Balb/cA and 129/Ola	E10.5–E15.5	Defects in neural tube formation	[37]
Same as above	Balb/cA	E15.5	Hypoplasia of liver, thymus and spleen; impaired definitive hematopoiesis	[38,43,44]
Same as above	C3H/HeJ	E11.5	Defects in neural tube and heart formation	[38–42]
Gene trap (intron 2) in R1 ES cells (retrovirus)	C57BL/C6	At birth	Heart formation defects; leaky expression of Jarid2 in nervous system	[45,46]
Floxed (exon 3) by targeting in 129/Sv ES cells	C57BL/C6	Not applicable	Mutation of *Jarid2* alleles specifically in cardiomyocytes on day E7.5 (αMHC-Cre) does not result in embryonic lethality	[47]

**Table 2:** Reported Jarid2-interacting proteins and candidate target genes

Interacting protein	Method	Target gene(s)	Cell type	Refs
Nkx2.5 and Gata4	Overexpression followed by *in vivo* protein IP in fibroblasts	Anf	Primary cardiomyocytes	[55]
Rb protein	*In vitro* GST pull down using cardiomyocytes lysates	Cyclin D1	Primary cardiomyocytes	[56]
Mef2a	*In vitro* GST pull down using cardiomyocytes lysates	αMHC/Myh6	Primary cardiomyocytes	[57]
Zfp496	*In vitro* GST pull down using heart extracts	Unknown		[58]
G9a and GLP	Overexpression followed by *in vivo* protein IP in fibroblast	Cyclin D1	Fibroblast	[54]
Polycomb repressive complex 2	Mass spectrometry, gel filtration and *in vivo* protein IP in ES cells	High number of developmental regulators	Embryonic stem cells	[5–9]
